# Strategic approach of multifaceted antibacterial mechanism of limonene traced in *Escherichia coli*

**DOI:** 10.1038/s41598-021-92843-3

**Published:** 2021-07-05

**Authors:** Akshi Gupta, Ebenezer Jeyakumar, Rubina Lawrence

**Affiliations:** Department of Industrial Microbiology, Jacob Institute of Biotechnology and Bioengineering, Technology and Sciences, Sam Higginbottom University of Agriculture, Prayagraj (Allahabad), Uttar Pradesh 211007 India

**Keywords:** Drug discovery, Microbiology

## Abstract

Antibacterial potential of Limonene against Multi Drug Resistant (MDR) pathogens was studied and mechanism explored. Microscopic techniques viz. Fluorescent Microscopy (FM), Scanning Electron Microscopy (SEM), and Transmission Electron Microscopy (TEM) indicated membrane disruption, cellular leakage and cell death of *Escherichia coli* (*E. coli*) cells when treated with limonene. Leakage of intracellular proteins, lipids and nucleic acid confirmed membrane damage and disruption of cell permeability barrier. Further, release of intracellular ATP, also suggested disruption of membrane barrier. Interaction of limonene with DNA revealed its capability in unwinding of plasmid, which could eventually inhibit DNA transcription and translation. Differential expression of various proteins and enzymes involved in transport, respiration, metabolism, chemotaxis, protein synthesis confirmed the mechanistic role of limonene on their functions. Limonene thus can be a potential candidate in drug development.

## Introduction

Treatment of infectious diseases is becoming a challenge for clinicians due to an alarming proportion of antibiotic resistance throughout the world accounting for significant morbidity and mortality. Increasing demands for new antibiotics are not being addressed adequately by pharmaceutical industries. Alternative therapeutic approach of age-old herbal knowledge to exploit secondary plant metabolites as potential antimicrobial agents could help in preparation of novel medicaments. Secondary metabolites of plants such as phenolics, alkaloids, saponins and terpenes are known to help and maintain homeostasis in their environment^1^. In comparison to currently used synthetic antibiotics, these phytochemicals are known to have negligible adverse reaction with potent antimicrobial property. One of the important constituents of plant essential oil is limonene belonging to the cyclic monoterpene hydrocarbon family, with hydrophobic nature. Limonene is generally recognised as safe (GRAS) and used as a fragrance and flavouring agent and hence is found in products ranging from cosmetics to beverages and ice-creams. It is believed to be a potential bioactive compound since it possesses various properties like antioxidant, anti-inflammatory, anticancer, insecticidal etc. With wide range of uses in culinary and therapeutic properties, limonene can be effectively exploited as an antibacterial agent. In order to explore natural compounds as medicine in a much more effective way, the science of its antibacterial property and the mechanism of action should be established to find the plausible role of the compound against various microorganisms. Previous studies on antibacterial mechanism of terpenes and their derivatives have demonstrated sub-lethal injury in the outer and cytoplasmic membranes as a plausible mechanism of *E. coli* cell death^2,3^. However, the sequence of events that lead to the cell death from the point of attachment/exposure to limonene is not documented till date. The present study thus provides an insight of various consecutive changes that occur in the *E. coli* cells on exposure to limonene causing cell injury eventually resulting in cell death.

## Results

Limonene was found to exhibit significant inhibitory activity being more effective against Gram positive bacteria with Zones of Inhibition ranging from 26.00 mm for *Bacillus subtilis* to 40.00 mm for *Clostridium perfringens* (P < 0.001). Among the Gram-negative bacteria maximum activity was recorded against *E. coli*, and *Helicobacter pylori* with zone of inhibition corresponding to 21.67 mm and 29.00 mm respectively while *Campylobacter jejuni*, *Pseudomonas aeruginosa* and *Salmonella typhi* were found to be resistant towards Limonene (Table 1).

**Table 1:** Effect of compounds from natural products against selected bacterial pathogens.

Organism	Zone of Inhibition (mm)* ± S.D
**a. Gram positive**	
*Staphylococcus aureus*	26.33 ± 0.57
*Staphylococcus aureus* (ATCC 25,923)	28.00 ± 1.00
*Listeria monocytogenes*	35.00 ± 1.00
*Bacillus cereus*	31.00 ± 2.00
*Bacillus subtilis*	26.00 ± 2.00
*Clostridium perfringens*	40.00 ± 1.00
*Streptococcus pyogenes*	30.00 ± 1.00
**b. Gram negative**	
*Escherichia coli*	21.67 ± 2.08
*Escherichia coli* (ATCC 25,922)	23.00 ± 1.00
*Campylobacter jejuni*	00.00
*Vibrio cholera*	15.00 ± 1.00
*Pseudomonas aeruginosa*	00.00
*Shigella dysenteriae*	16.33 ± 1.15
*Salmonella typhi*	00.00
*Helicobacter pylori*	29.00 ± 1.00

*Includes 5 mm well size.

Minimum Inhibitory Concentration (MIC) of limonene against reference strain of *E. coli* corresponded to 16 µg/mL with a twofold increase in the Minimum Bactericidal Concentration (MBC) value (Table 2). Addition of Mg^2+^ or Ca^2+^ (10 mM) resulted in twofold increase of MIC value of limonene and a 128-fold increase in the MIC of colistin used as standard. (Table 3).

**Table 2:** Minimum inhibitory concentration (MIC) and minimum bactericidal concentration (MBC) of limonene against reference strains.

Test bacteria	Minimum inhibitory and bactericidal concentrations (µL/mL)		
	IMIC	FMIC	MBC
*E. coli* ATCC 25922	16	16	32

*IMIC* initial minimum inhibitory concentration (after 24 h); *FMIC* final minimum inhibitory concentration (after 48 h); *MBC* minimum bactericidal concentration.

**Table 3:** Effect of divalent cation salt solutions on the bactericidal activity of Limonene.

Organism	MIC (µL or µg/mL)					
	Without Mg^2+^ or Ca^2+^		With 10 mM Mg^2+^		With 10 mM Ca^2+^	
	Limonene	Colistin	Limonene	Colistin	Limonene	Colistin
*E. coli*	16	8	32	1024	32	1024

**Figure 1:**
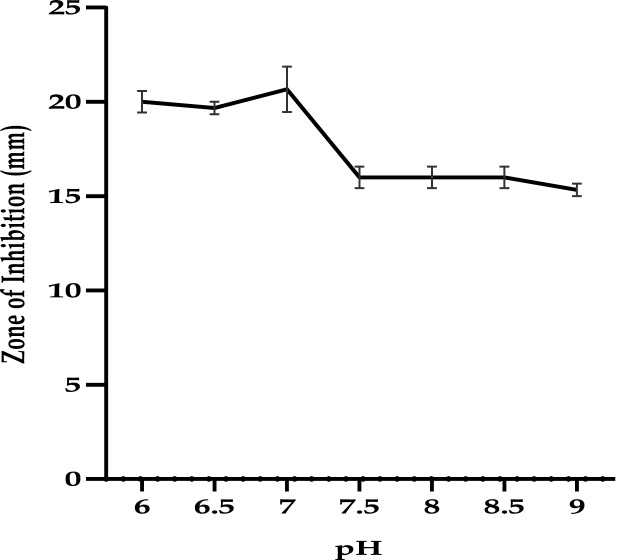
Activity of limonene (1XMIC) at different pH.

**Figure 2:**
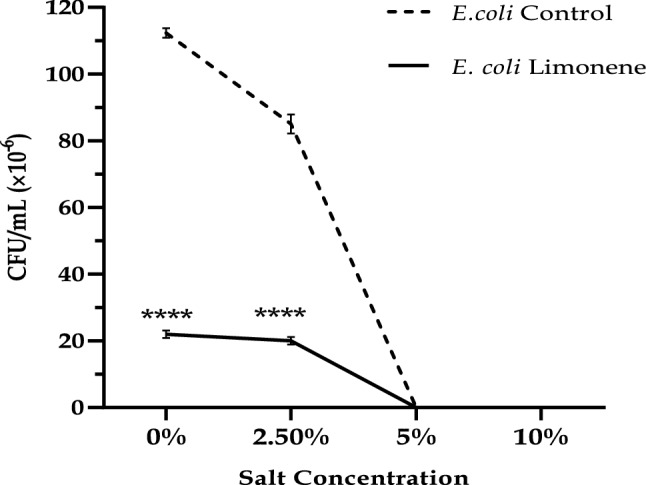
Salt tolerance of *Escherichia coli* treated with limonene (1XMIC).

Limonene was found to exhibit antibacterial activity against *E. coli* in the wide range of pH tested (pH 6.0–9.0). Limonene was found to have maximum activity in terms of zones of inhibition at neutral pH (optimal growth pH). Limonene was also found effective against *E. coli* in the acidic conditions with pH 6.0–6.5. With the increase in pH a decreasing trend in the zone of inhibition was observed against the test pathogen with least values corresponding at pH 9.0 (Fig. 1). Significant reduction (80.36%) in the CFU/mL of *E. coli* cells exposed to limonene at MIC concentration was observed with increasing NaCl concentrations with total inhibition at 5% concentration. However, the untreated *E. coli* cells demonstrated salt tolerance up to 5% NaCl concentration (P < 0.0001) (Fig. 2).

**Figure 3:**
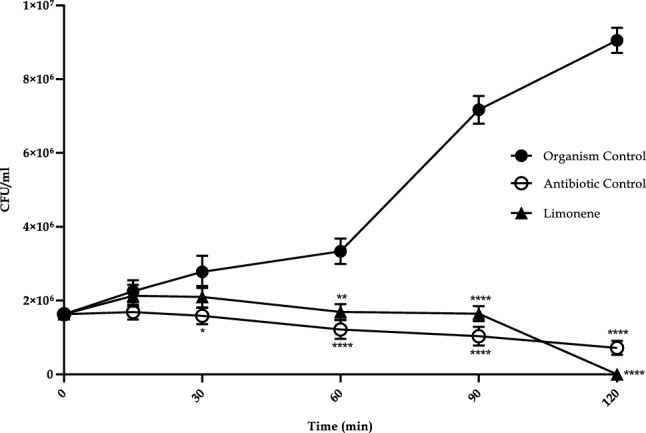
Time Kill Curve of limonene (1XMIC) against *Escherichia coli.*

A significant reduction in log cfu/mL of *E. coli* was observed when treated with limonene. Seven percent reduction in *E. coli* was recorded within 15 min which increased to 77% at 1.5 h and 100% (6 log reduction) at 2 h. The rate of killing of *E. coli* with limonene was half that of the standard antibiotic used as positive control (P < 0.0001) (Fig. 3).

**Figure 4:**
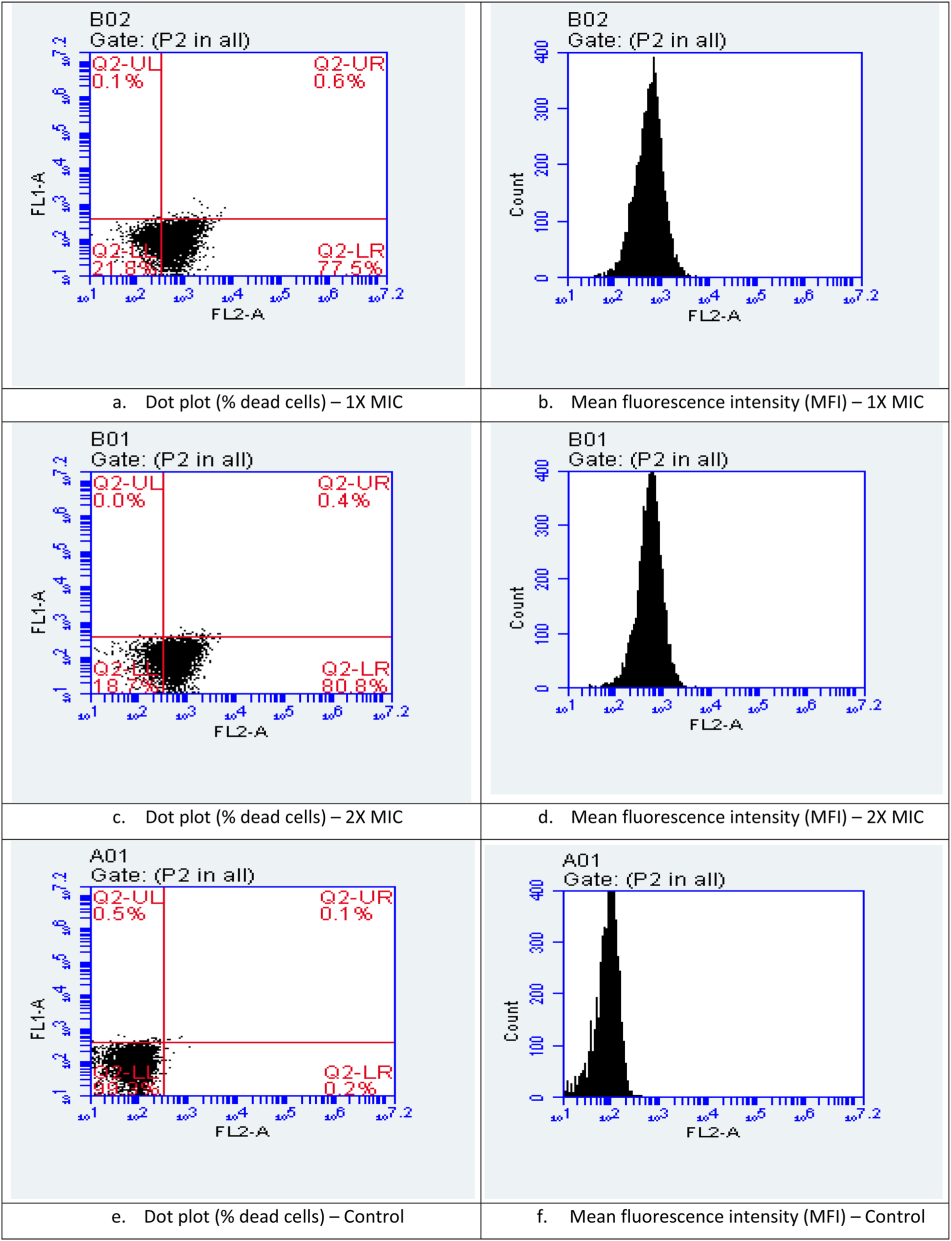
Membrane permeability of *Escherichia coli* cells at 1X MIC (**a**,**b**), 2X MIC (**c**,**d**) of Limonene, control: without limonene (**e**,**f**).

**Figure 5:**
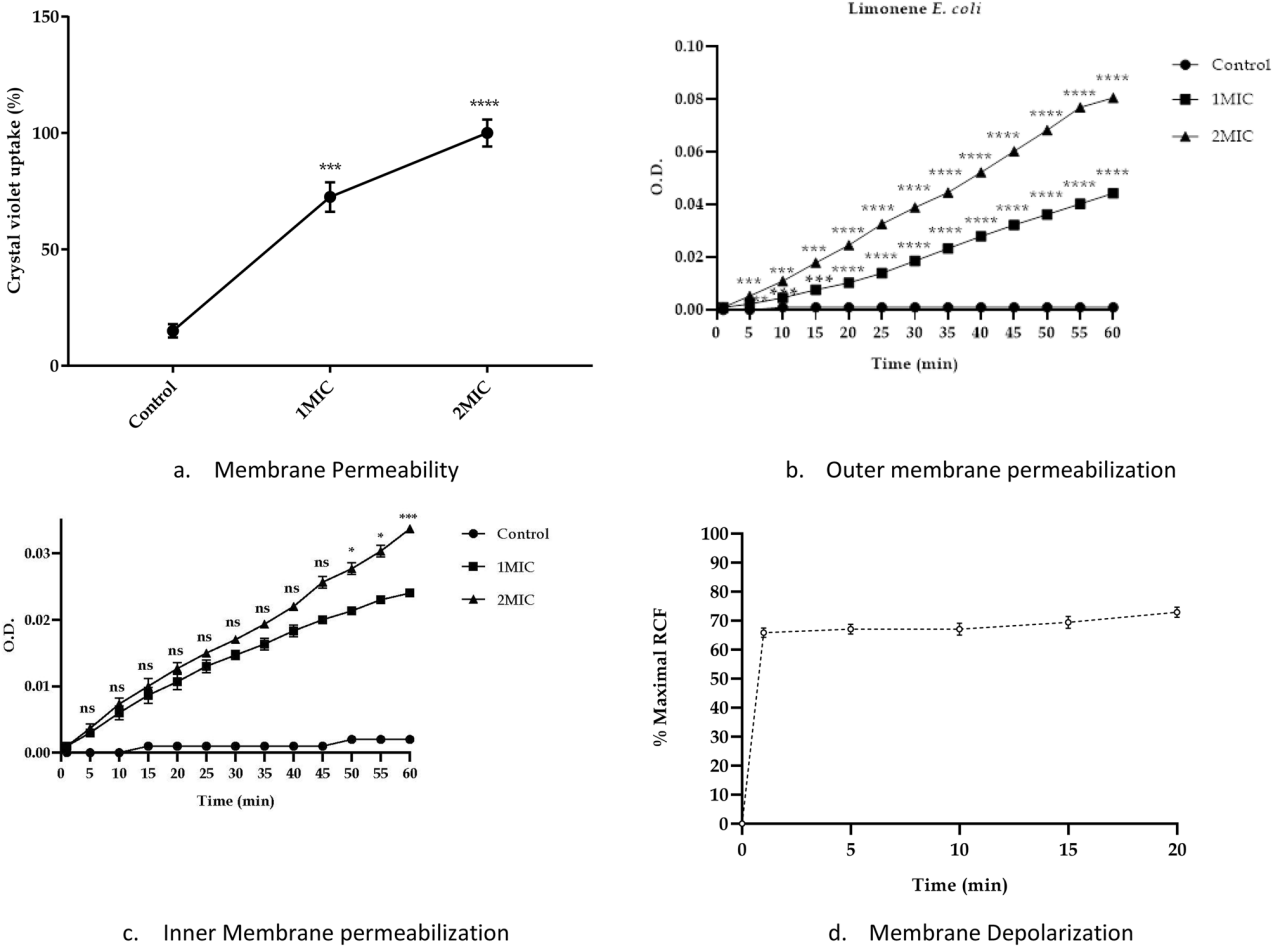
Effect of limonene on (**a**) membrane permeability, (**b**) outer membrane permeabilization, (**c**) inner membrane permeabilization, (**d**) membrane depolarization in *Escherichia coli* [****P ≤ 0.0001; ***P ≤ 0.001; **P ≤ 0.01; *P ≤ 0.05].

**Figure 6:**
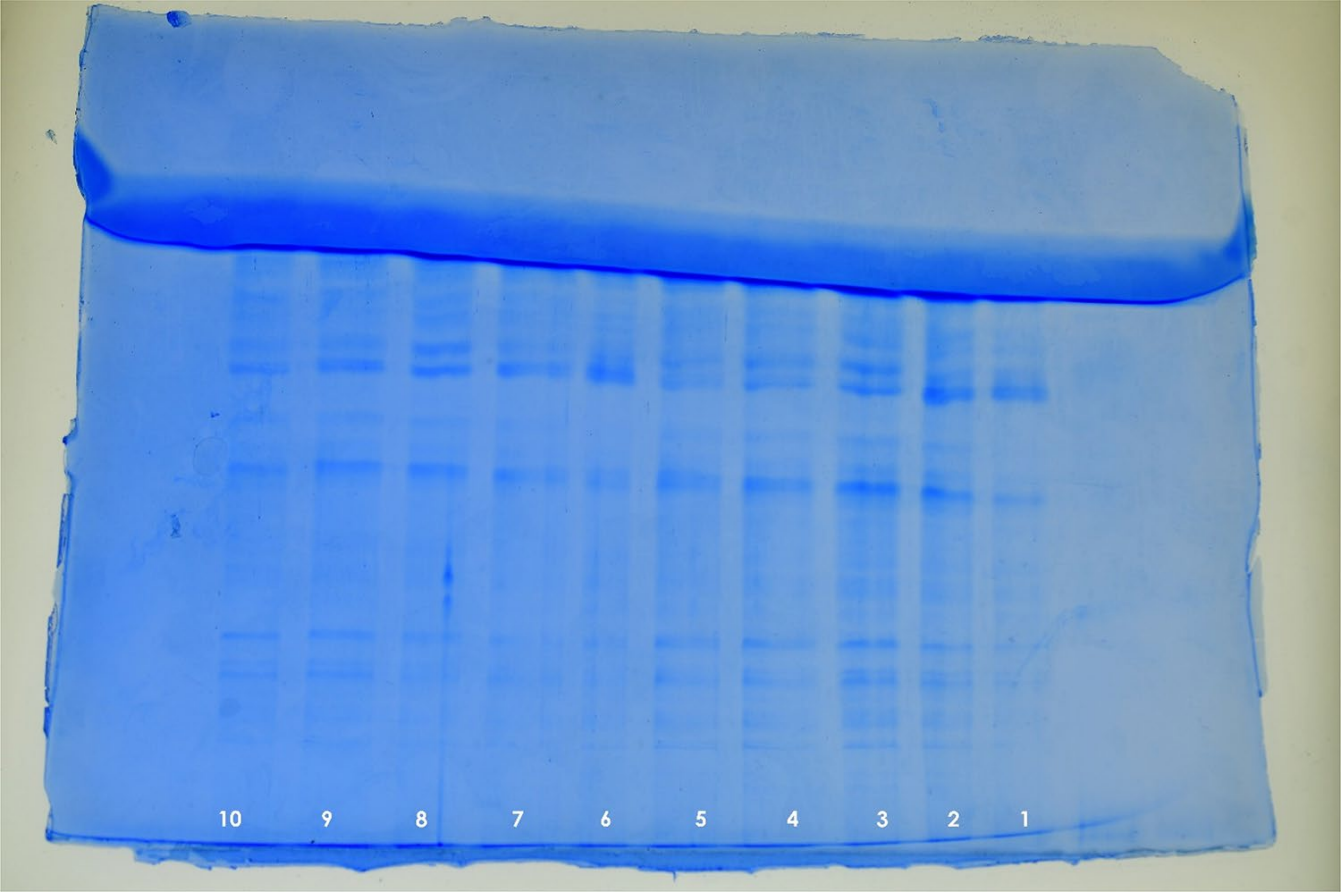
SDS-PAGE of proteins released from *Escherichia coli* into supernatant on treatment with limonene at 1XMIC and 2XMIC concentration. Lane 1 to 5 1XMIC (Lane 1: 0 min|Lane 2: 30 min|Lane 3: 60 min|Lane 4: 90 min|Lane 5: 120 min). Lane 6 to 10 2XMIC (Lane 6: 0 min|Lane 7: 30 min|Lane 8: 60 min|Lane 9: 90 min|Lane 10: 120 min).

**Figure 7:**
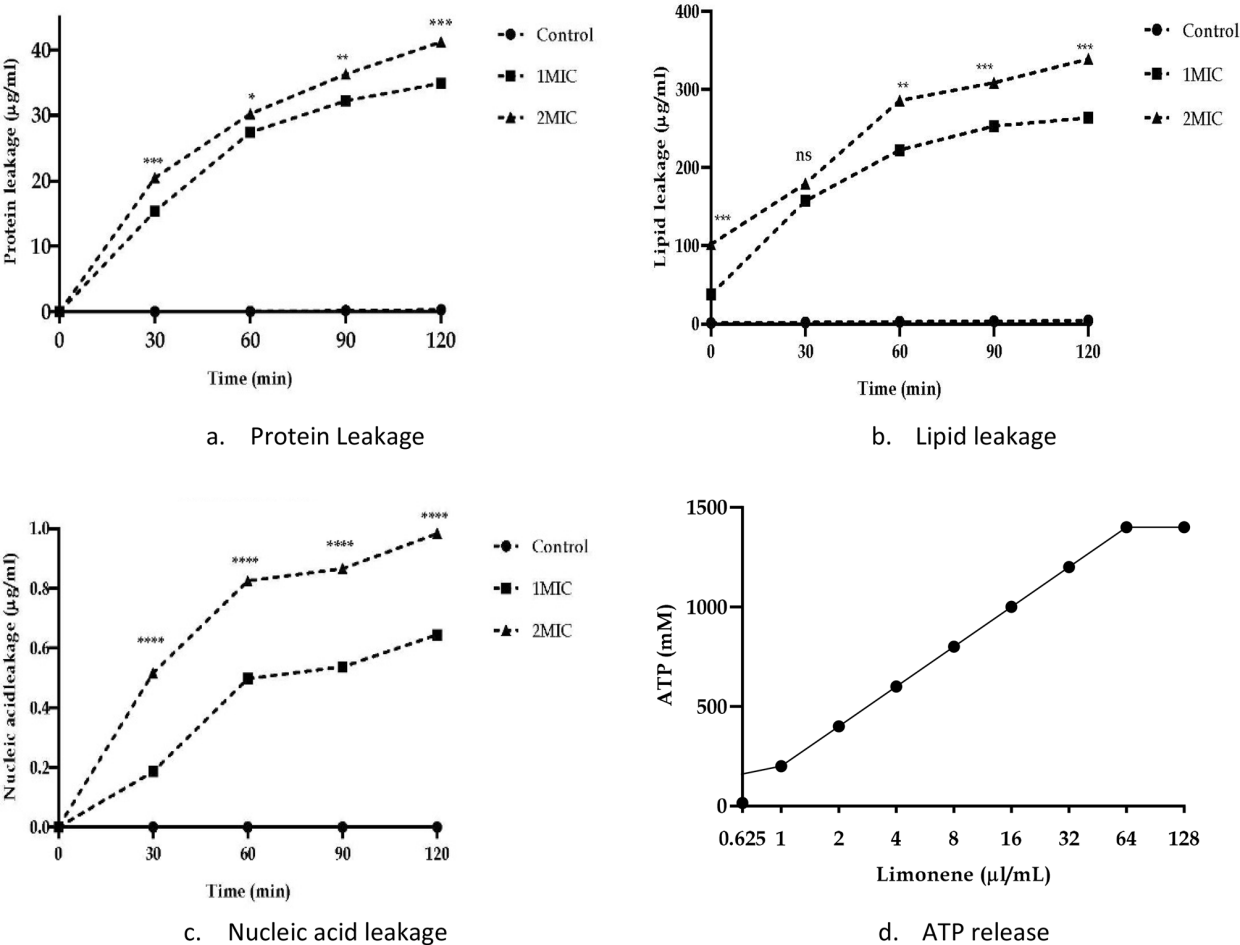
Effect of limonene on leakage of (**a**) protein, (**b**) lipid, (**c**) nucleic acid and (**d**) ATP in *Escherichia coli.*

**Figure 8:**
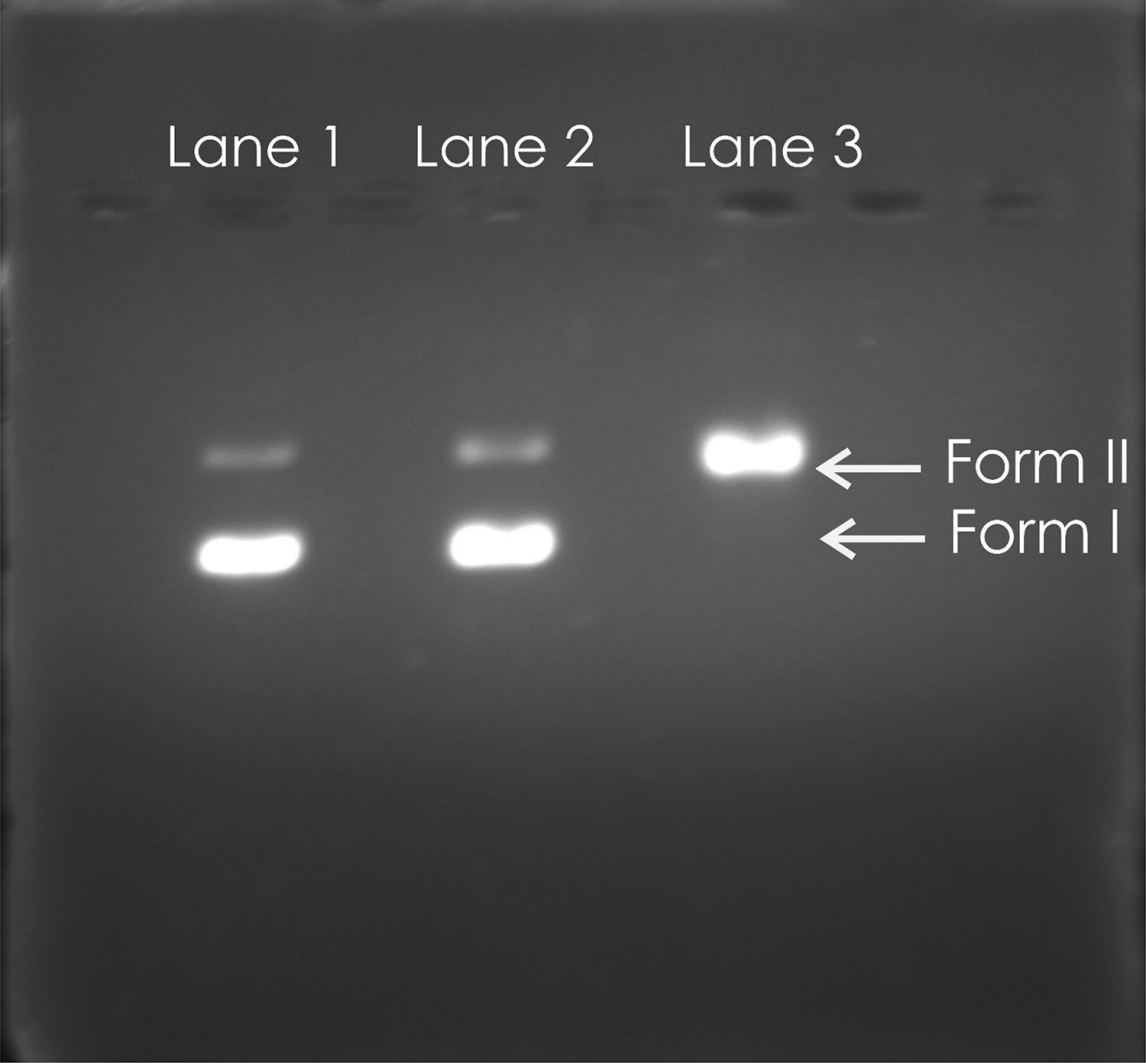
Modification of gel electrophoretic mobility of pUC19 plasmid DNA after treatment with Limonene. Lane 1- Negative control, Lane 2- Pure plasmid, Lane 3- Limonene (1XMIC).

An increased membrane permeability of *E. coli* cells treated with limonene at 1X and 2XMIC concentrations was observed on the basis of mean fluorescence intensity recorded using propidium iodide in flow cytometry as compared to control (Fig. 4). Similarly, an increase in the crystal violet uptake of 72.5% with 1XMIC and 100% uptake at 2XMIC compared to untreated cells (15%) (P < 0.001) confirms the alteration in membrane permeability (Fig. 5a). A significant increase in O. D. values due to alteration of outer membrane permeability with respect to entry of nitrocefin inside the *E. coli* cells treated with limonene as compared to control (P < 0.001). Thus, a dose dependent increase in alteration in outer membrane permeability of *E. coli*, with increase in time was observed (Fig. 5b). Using ONPG, the permeability of inner membrane was found to be significantly affected in the *E. coli* cells treated with limonene at 1XMIC and 2X MIC as compared to control (P < 0.0001) (Fig. 5c). Further, a time-dependant membrane depolarization in *E. coli* cells treated with limonene (@MIC) was observed corresponding to 65.88% RCF within 1 min which further increased to 84.71% after 240 min. (Fig. 5d). With increasing time (0–120 min), exposure of *E. coli* to limonene showed a proportional increase in the protein leaked in to the medium as evident by the protein bands observed in SDS-PAGE (Fig. 6). An increase in protein leakage corresponding to 15.67 µg/mL to 35.27 µg/mL and 20.9 µg/mL to 41.89 µg/mL at MIC and twice MIC respectively was recorded (P < 0.001) (Fig. 7a). Similarly, leakage of lipid with values corresponding to 342.55 µg/mL (2XMIC) and 270.21 µg/mL (MIC) was estimated (P < 0.0001) (Fig. 7b). Leakage of 260 nm absorbing cellular material comprising of nucleic acids, metabolites and ions was demonstrated with values ranging between 0.192–0.650 at MIC and 0.529–0.985 at 2XMIC (P < 0.0001) (Fig. 7c). With increasing concentration of limonene from 0.0625 to 128 µL/mL resulted in relative increase in intracellular release of ATP in *E. coli* with values corresponding to 15 nM to 1400 nM respectively suggesting the disruption of the membrane barrier (Fig. 7d). Electrophoresis of plasmid DNA resulted in covalently closed circular form I and Nick circular form II bands visible in the gel; form I migrating faster than form II. On treating with limonene, a single broad band at the region of form II was visible suggesting the effect of the compound on covalently closed circular and supercoiled plasmid to form nick circular form II. This supports the capacity of the compound in causing conformational changes in DNA helix that may affect the stability and its subsequent susceptibility to degradation. (Fig. 8).

**Figure 9:**
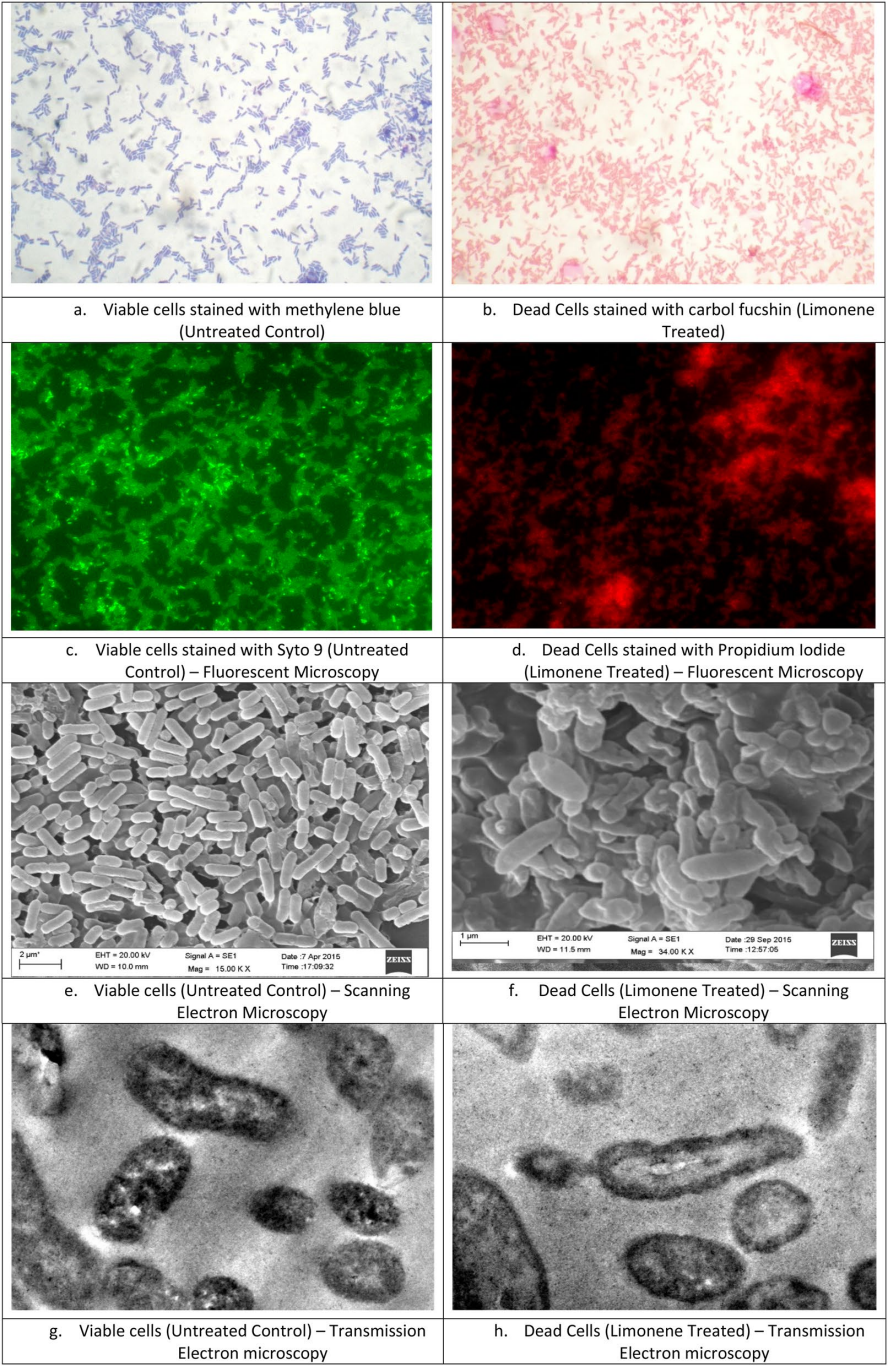
Micrographs of untreated control and limonene treated (1XMIC) *E. coli* cells using Compound microscopy (**a**,**b**) fluorescent microscopy (**c**,**d**) Scanning electron microscopy (**e**,**f**) Transmission Electron Microscopy (**g**,**h**).

The effect of limonene on cell damage leading to death of *E. coli* was further authenticated using microscopy. On the basis of viability test, limonene treated *E. coli* cells appeared red in color due to carbol fuchin, while untreated cells appeared blue due to methylene blue staining suggesting complete cell death in the treated group (Fig. 9a,b). Using propidium iodide, the bacterial cells of test organisms treated with 1XMIC concentration of limonene showed a significant increase in PI associated fluorescence (Fig. 9c,d).

The treatment of *E. coli* with limonene resulted in significant morphological changes as observed through SEM and TEM studies. The untreated cells as visualized through SEM were found to be intact and the TEM images showed unanimous electron density suggesting that cells are in a normal condition without environment disturbance.

The Scanning Electron Microscopic studies showed, indentation and damaged outer membrane of *E. coli* on treatment of the organism with 16 µL/mL of Limonene (Fig. 9e,f). The observation was further confirmed through Transmission Electron Microscopy which revealed altered and disrupted cell membrane (Fig. 9g,h). Some cells were irregularly shaped and parts of the cell were broken leading to leaching out of cell constituents. Further electron dense granules around the cell wall and electron light region in the centre of the cell was also observed.

**Figure 10:**
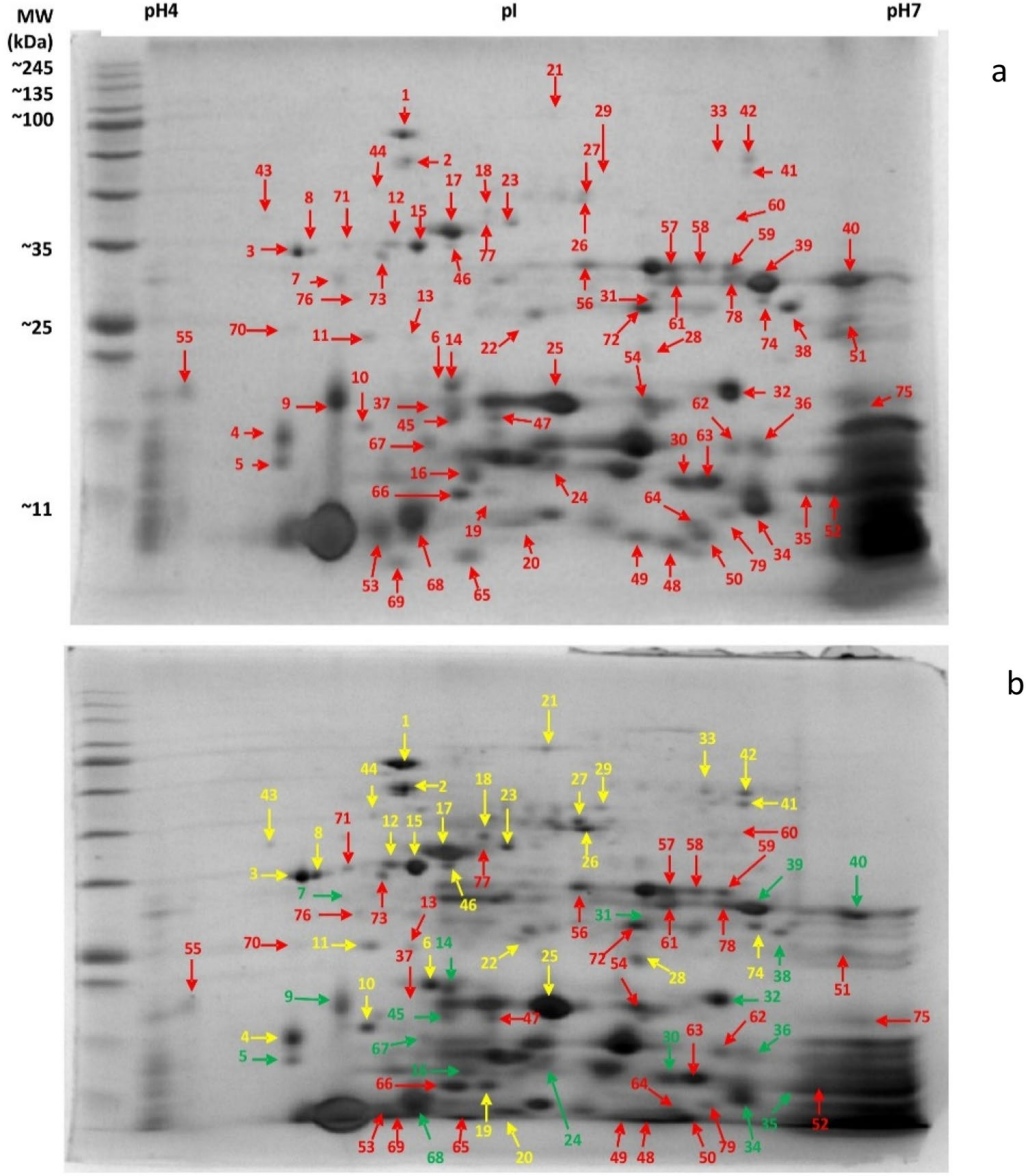
The 2D gel protein image of limonene treated (1XMIC) *E. coli* cells. (**a**) Control, (**b**) treatment; yellow arrow = upregulate | green arrow = downregulate | red arrow = no change (PDQuest Advanced 2-D Gel Analysis software version 8.0.0 (Bio-Rad, Hercules, CA, USA) https://www.bio-rad.com/en-in/sku/1709630-pdquest-advanced-2-d-analysis-software?ID=1709630).

The protein expression accounted ≥ 1.5 fold change (47 spots) as compared to control. Among them 27 spots were identified with 17 upregulated and 10 downregulated proteins (Fig. 10; Table 4).

**Table 4:** Differently expressed proteins of *E. coli* after limonene treatment.

Spot no:	Upregulated proteins	Spot no:	Downregulated proteins
1	Chaperone protein DnaK, Heat shock 70 kDa protein	7	Chemotaxis protein CHEZ
2	30S ribosomal protein S1	9	Biotin carboxyl carrier protein of acetyl-CoA carboxylase
3	Flagellar hook-associated Protein 3	14	Protein yceI, Flags: Precursor
6	3-Keto-l-gulonate-6-phosphate decarboxylase ulaD	16	2-iminobutanoate, 2-iminopropanoate deaminases
10	Dihydrofolate reductase	31	Triosephosphate isomerase
15	Transaldolase B	36	50S ribosomal protein L9
17	Malate dehydrogenase	39	Hydrogenase isoenzymes nickel incorporation protein hypB
18	Probable hexulose-6-phosphate synthase Phosphoserine aminotransferase	40	Molybdate-binding periplasmic protein
19	Xanthine phosphoribosyltransferase	45	DNA protection during starvation protein
21	Methionyl-tRNA synthetase	67	Outer membrane protein X
26	Diaminopimelate decarboxylase		
27	Glutathione reductase		
28	Glyceraldehyde-3-phosphate dehydrogenase A		
29	3-isopropylmalate dehydratase		
43	Outer membrane protein OmpC (Porin)		
44	Trigger factor		

**Figure 11:**
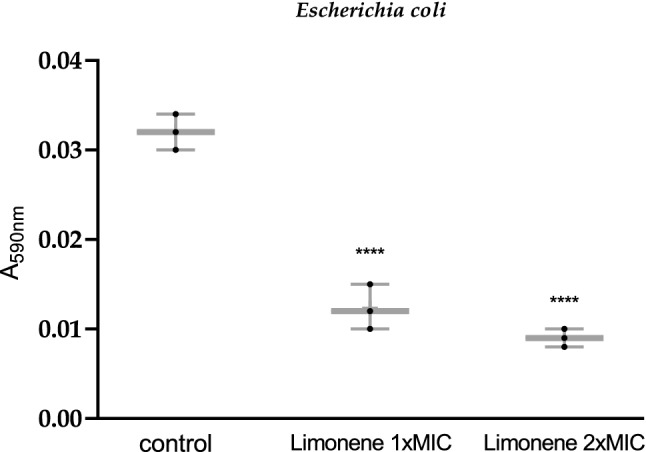
Biofilm inhibition assay of limonene (1X and 2XMIC) against *Escherichia coli*, graph is shown in a box and whisker format. Boxes range from the minimum to maximum percentile and are intersected by median line. Whiskers extending below and above the box range from 1st to 90th percentile, respectively. All data points are also indicated individually.

There was a significant reduction in biofilm formation in limonene treated (1XMIC and 2XMIC) *E. coli* cells as compared to untreated control (Fig. 11).

## Discussion

Antibacterial property of limonene and its enantiomers has been well documented and is comparable with the present study findings^2^. Greater activity of limonene against Gram positives is also observed by other workers^3^. This phenomenon could be due to the relatively impermeable outer membrane that surrounds gram-negative bacteria. However, in the studies of Haiyan et al.^4^, gram-negatives are reported to be more sensitive to limonene which could be related to different targets of action. Variation in MIC values of limonene as compared to the present work has been observed which could be attributed to the strains used in the study and different experimental conditions adopted^3^. Increase in MIC values in the presence of Ca^2+^ and Mg^2+^ ions observed could be due outer membrane stabilization. The divalent cations forms complexes with antibacterial agents that are too bulky to be able to cross the porin channels present in bacterial membrane thus altering the membrane properties and subsequently preventing damage by antibacterial agents^5^. Limonene may thus act as membrane active molecule targeting the bacterial membrane by interacting with negatively charged phosphate group in Lipopolysaccharide (LPS).

The ability of limonene to inhibit *E. coli* in an acidic environment as observed in the present study is of great importance in the treatment of infections caused on the sites having mildly acidic pH since *E. coli* utilizes energy to maintain their pH values within cell in an acidic environment^6^. Microorganisms are able to develop a series of mechanisms to sense, respond, resist and adjust to hyper osmotic stress like osmoregulation. However, this property was diminished in the presence of limonene to a greater extent which could be attributed to injured bacterial cell membrane, altering permeability and ability to osmoregulate^7^. Killing of *E. coli* by limonene within 2 h (log phase) classifies it under the fast-acting compound category of Friedman et al.^8^. The rate of killing is dependent on concentration of compound, duration of exposure, and bacterial strain used in the study^9^.

Penetration of crystal violet in the cell membrane of limonene treated *E. coli* cells is indicative of it being damaged or impaired^10^. Entry of nitrocefin inside the bacterial cell further confirms alteration in membrane permeability^11^ due to damage of outer membrane proteins that plays an important role in the formation of hydrophilic channels which selectively allows the uptake of required substances inside the cells^12^. Alteration in inner membrane permeability as detected by passage of ONPG confirms the capability of limonene in influencing membrane fluidity and permeability by influencing the membrane structure by changing the topology of membrane proteins^13^. As in the present study, more than 100-fold increase in the fluorescence of the dye after depolarization has also been reported by Winkel et al.^14^ suggestive of disruption of membrane integrity of bacterial isolates. Further penetration of propidium iodide is an indication of the presence of substantial, irreparable breaches in the membrane which leads to the cell death^15^.

As a consequence of membrane damage and disruption of cell permeability barrier due to limonene, leakage of cellular constituents viz protein, lipid and 260 nm absorbing material (nucleic acid) was observed which serves as a good indicator of compromised membrane integrity^16^.

As in the study, increased release of intracellular ATP, an indicator of membrane lesions, on treatment of test organism with bioactive agent of similar nature viz. eugenol^17^ and carvone^18^ has been reported. Further, binding of drug to the DNA causes various structural and conformational changes resulting in DNA damage and inhibition of DNA transcription and replication comparable with the present findings^19^.

Cellular and membrane integrity is considered as the main criteria for differentiation of live and dead cells. Propidium iodide (PI) intercalates with DNA^20^ following which fluorescence of PI increases by 20 to 30 folds as observed in the study. Further *E. coli* cells are reported to swell and disrupt due to cell surface damage, leading to dysfunction of cell membrane and debris formation as observed via electron microscopy when treated with essential oils containing terpenes^21^.

The chemoattractant property of Limonene against gram-negative bacteria is similar to the study conducted on Eugenol by Devi et al.^10^. *E. coli* can actively swim with response to spatiotemporal chemical gradients and presence of exopolysaccharides viz., glucose, n-acetyl-galactosamine, fucose, and mannose at high concentrations have been reported to act as chemoattractant thus explaining the chemotaxis-assisted formation of bacterial clusters.

**Figure 12:**
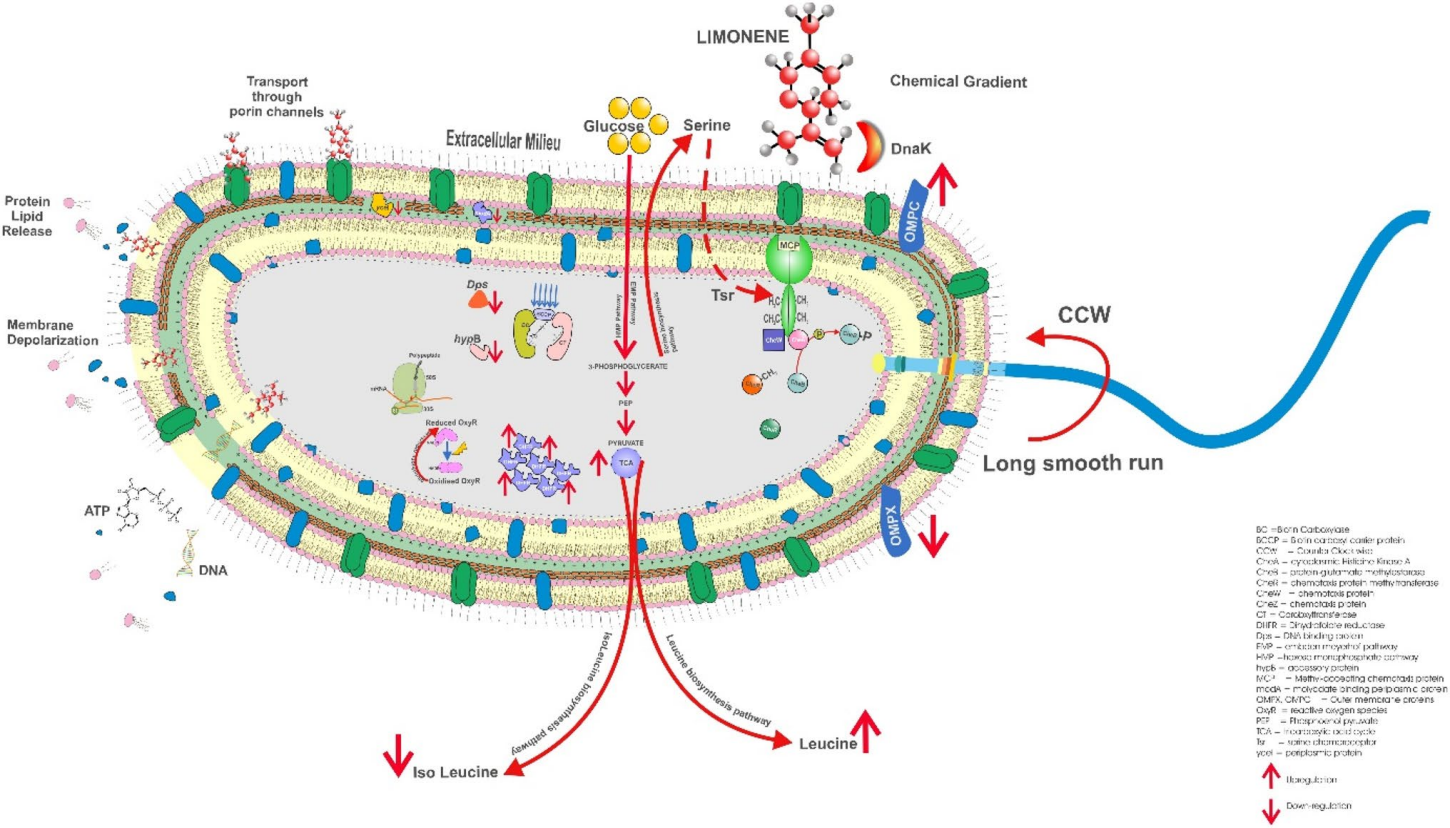
Antibacterial mechanism of Limonene against *Escherichia coli.*

Overexpression of phosphoserine aminotransferase indicates production of serine and presence in the extracellular fluid signals transmembrane chemoreceptor *tsr* (methyl accepting protein, MCP) in the periplasmic space to binds with limonene; CheR (methyltransferase), and CheB (methylesterase) maintains methylated state and CheA (kinase) autophosphorylates CheB to continue this process. Downregulation of chemotaxis protein CheZ also confirms that at this stage CheY is not phosphorylated, hence cell mobility towards chemical gradient can be predicted as smooth and long run. Overexpression of flagellar hook-associated protein 3 indicates an increase in flagellar dependant motility in presence of limonene. Enzymes involved in phosphotransferase system (PTS) were upregulated and responsible for glucose molecule to gain entry in to the cell. Further, upregulation of 3-keto-l-gulonate-6-phosphate decarboxylase ulaD, hexulose-6-phosphate synthase, glyceraldehyde-3-phosphate dehydrogenase A involved in glucose metabolism (Hexose Monophosphate Pathway and Embden Meyerhoff Parnas Pathway) leading to formation of pyruvate via 3-phosphoglycerate was observed. However, enzyme involved in isomerization of glyceraldehye-3-phosphate to dihydroxy acetone phosphate was repressed. Overexpression of certain enzymes involved in metabolism of pyruvate via TCA cycle was also an indication of the functionality of this pathway. Upregulation of enzyme involved in synthesis of leucine (3-isopropyl malate dehydratase) and downregulation of isoleucine (2-iminobutanoate) was also observed. Further, pathway of serine biosynthesis involved in flagellar movement was traced down from 3-phosphoglycerate with overexpression of phosphoserine aminotransferase. Diaminopimeleate decarboxylase involved in synthesis of essential amino acid lysine was also found to be upregulated. Lysine is a promising antibacterial target and is also important in the synthesis of peptidoglycan cell wall, housekeeping protein and virulence factors of bacteria. Heterocyclic compounds are also potential inhibitors of DNA Protein Crosslinks (DPC) enzyme which eventually affects cell wall synthesis and virulence nature of the organism. Bacterial cells activate stringent response during stress and starvation which leads to down-regulation of energy-requiring processes related to growth and upregulation process associated with survival and stress response. This mechanism involves synthesis of hyperphosphorylated nucleotides guanosine pentaphosphate and guanosine tetraphosphate referred as ‘alarmone’ from GTP/GDP and ATP as a response to starvation or stress and alerts the bacterial cellular machinery to conserve available resources. Xanthine phosphoribosyltransferase, a key enzyme involved was upregulated in *E. coli* in the presence of limonene suggesting the cell triggering Purine salvage pathway as defence against stress. Overexpression of glutathione reductase plays a role in defence mechanism, protecting the cell against oxidation, by maintaining a pool of reduced glutathione. This in turn may serve to maintain reduced state of cellular proteins since the oxidative stress has been reported to damage DNA, membrane and proteins in *E. coli*^22^. Similarly, *E. coli* cells upregulate methionyl-tRNA synthetase (MRS) which under oxidative stress, charges Met to noncognate tRNAs (Met-misacylation) at high frequency resulting in suppression of global translation^23^. Simultaneous overexpression of 30S ribosomal protein S1 and downregulation of 50S ribosomal protein L9 indicates a clear disruption of translation process in protein synthesis. This phenomenon seems to serve as a defense mechanism against Reactive Oxygen species (ROS) mediated damage at the cost of translational fidelity. Overexpression of OmpX present in outer membrane of *E. coli* decreases OmpF and OmpC porins resulting in antibiotic resistance. The reverse was observed with downregulation of OmpX and upregulation of OmpC suggesting sensitivity of *E. coli* towards limonene. OmpX facilitates invasion of host cells and also has a role in resistance against human compliment system. Its downregulation suggests loss of invasiveness and cell immunity. DnaK an allosteric essential chaperon protein has strong affinity for hydrophobic peptides. Limonene being hydrophobic in nature can mimic these hydrophobic peptides and can get easily transported through porin channels with the help of overexpressed chaperon proteins. Overexpression of dihydrofolate reductase (DHFR) observed in limonene treated *E. coli* could be responsible for toxic metabolic imbalance, triggered by interaction with several functionally related enzymes. The Biotin carboxyl carrier protein of acetyl CoA carboxylase (ACC) catalyzing the first step of fatty acid synthesis was downregulated indicating that limonene is also targeting cell wall inhibition of *E. coli*. Yao and Rock^24^ also suggested inhibition of enzymes involved in fatty acid synthesis as desirable target for antibiotic discovery. yceI, a periplasmic protein is responsible for various physiological functions like protection against harmful external factors, uptake, salvage and chemotaxis of various nutrients. The small size of limonene enables the molecule to diffuse through the outer membrane challenging the periplasmic protein yceI which was downregulated in the study thus hindering their performance. Thus, it is also expected to affect isoprenoid biosynthesis eventually affecting its function in cell respiration, signalling, growth regulation and membrane biosynthesis. HypB (accessory protein) involved in maturation of all hydrogenase enzymes and permitting bacteria to use hydrogen as energy source for growth was downregulated in limonene treated *E. coli* cells. Further, downregulation of molybdate binding periplasmic protein adversely affects the molybdenum uptake affecting enzyme activity and molybdate transport machinery. Bacterial deficit in DNA binding protein (Dps protein) from starved cells show dramatically increased mortality rates when exposed to any of the stresses, including starvation, oxidative stress, metal toxicity or thermal stress. Downregulation of Dps in the presence of limonene in *E. coli* could be a plausible reason for cell undergoing stress leading to death (Fig. 12).

The damage caused in the cell wall, cytoplasmic membrane or surface proteins could be attributed to its inability for biofilm formation^3^. Limonene being a terpene, could also influence fatty acid composition of bacterial cell membrane affecting the hydrophobicity of the cell ultimately leading to eradication of biofilm.

## Methods

### Antibacterial activity, MIC and MBC of limonene

Limonene was previously isolated and purified and found to have potent antibacterial activity in our laboratory. However, for the present study the same has been obtained from (Sigma Aldrich) for the sake of its purity (96%). Thirteen multi-drug resistant clinical bacterial pathogenic isolates were tested for their sensitivity towards limonene using agar well diffusion method^6^. The inoculated plates were incubated at 37 ± 1 °C and zones of inhibition recorded after 24 h. The results were interpreted as per the criteria recommended by Chattopadhyay et al.^25^.

The minimum inhibitory concentration (MIC) of limonene was determined by broth dilution technique using resazurin microtiter plate assay^26^. Overnight broth cultures of *E. coli* in Muller Hinton Broth with a cell density adjusted to 1 × 10^6^ CFU/mL was added to microtiter plate containing serially diluted compound with resazurin 0.02% as indicator. Following incubation at 37 ± 1 °C, MIC values (Initial MIC at 24 h and Final MIC at 48 h) were recorded and the bactericidal concentration determined by inoculating loopful cultures from MIC wells on to Muller Hinton Agar plates.

Since *E*. *coli* (ATCC 25922) is a control strain for antimicrobial susceptibility testing recommended by CLSI (Clinical and Laboratory Standards Institute), the same has been used to determine MIC, MBC and mode of action of limonene in the present study.

### Effect of divalent cations on activity

Resazurin micro titre plate assay used to determine MIC values was performed to study the effect of divalent cations on antibacterial activity of limonene. Muller Hinton broth supplemented with different concentrations of divalent cations (Magnesium chloride / Calcium chloride) and limonene were serially diluted and bacterial suspension with 0.02% resazurin was added in each well. On the basis of color change, lowest concentration of the compound that brings about the change was taken as MIC value^27^.

### pH sensitivity assay

Overnight broth cultures of selected bacteria with different pH ranges (5.5, 6.0, 6.5, 7.0, 7.5, 8.0, 8.5 and 9.0) were swabbed on Muller Hinton Agar plates with corresponding pH. Antibacterial activity was determined by agar well diffusion assay as described in the previous section and zone of inhibition (mm) recorded after 24 h of incubation^28^.

### Loss of salt tolerance capacity

Overnight broth culture of test organism was treated with 1X MIC of limonene for 1 h at 37 ± 1 °C followed by inoculation of nutrient agar plates supplemented with different concentrations of NaCl (0%, 2.5%, 5.0%, and 10%). Untreated bacterial culture used as control was compared with treated plates and results expressed in terms of Log_10_ CFU/mL^29^.

### Time kill assay

The time kill assay of limonene treated *E. coli* cells was sampled at 0, 0.25, 0.5, 1, 1.5, 2, 2.5, 3, 3.5, 4, 5….0.24 h; pour plated with Muller Hinton Agar and incubated at 37 ± 1 °C for 24 h. The values were expressed as Log_10_ CFU/mL^30^.

### Membrane permeabilization, depolarization and disruption

*Escherichia coli* cells were harvested from overnight broth culture, washed thrice and resuspended in PBS to a concentration of 0.1 at OD 625. The cell suspension was treated with limonene (1XMIC and 2XMIC) and alternation in membrane permeabilization recorded.

First set of treated suspension was exposed to 10 μg/mL of crystal violet and observations read at OD 590^10^. Second set exposure of treated cell suspension to 30 μM nitrocefin was read at 1 min interval till 1 h at 486 nm (2010). Flow cytometric analysis using propidium iodide for staining of treated cells was recorded using 488 nm laser to detect red coloured fluorescence and analyzed using the BD AccuriC6 software for third set of treatments^31^.

Inner membrane permeabilization was studied by mixing 2.5 mM ONPG and recording at 420 nm at 1 min interval for 1 h^32^.

Membrane depolarization was evaluated by adding 3, 3-dipropylthiadicarbocyanine iodide (3 μM) to the treated cells and measuring fluorescence at excitation / emission wavelengths of 660/675 nm respectively^32^. Using SDS-PAGE the protein obtained by disruption of bacterial membrane due to limonene (1XMIC and 2XMIC) was also detected at 30 min interval from 0 to 120 min^10,33^.

### Protein, lipid and nuclei acid leakage

Leakage of protein and lipid^34^ from *E. coli* cell suspension obtained after harvesting overnight broth culture diluted to 0.1 at OD 625 and treated with limonene (1XMIC and 2XMIC) was recorded at 595 and 525 nm respectively. Release of cellular material was estimated by centrifuging aliquots of cell suspension treated with limonene at 30 min intervals for at 13,400Xg 15 min and OD 260 of the supernatant was read as % release of extracellular UV absorbing material^11,16^.

### Measurement of ATP release

*Escherichia coli* cells treated with limonene at MIC and twice MIC values was aliquoted (100 µL) at different time intervals and Luciferin-luciferase reagent (Promega, USA) was added (50 µL). Luminescent signal was read using Titertek Berthlod Sirius L Luminometer for 5 s^32^.

### DNA and compound interaction assay

Interaction of limonene with bacterial DNA was investigated using protocols of Oztrurk et al.^19^. Plasmid (1μL) was added to different concentration of limonene in a tube containing TE buffer (pH 7.4) and incubated at 37 ± 1 °C for 24 h in dark. Then 10 μL of the mixture was mixed with gel loading buffer and run on 1% agarose gel containing ethidium bromide and visualized using UV transilluminator (302 nm).

### Alteration in Cellular morphology

Overnight broth culture of *E. coli* cells was harvested at 6000 rpm for 10 min, washed with PBS (1X) and concentration adjusted to 0.1 at OD_625_. Following treatment with limonene at MIC and twice MIC values the cellular viability of the test organism was observed following staining with methylene blue and carbol fucshin under compound microscope (100X)^35^.

Treated cells were further stained with Syto 9 (5 mM) and Propidium iodide (1 mg/mL) and observed under fluorescent microscope with blue and green filters^36^. Morphological changes of treated cells were also studied using Scanning Electron Microscope (SEM)^37^ and Transmission Electron Microscopy (TEM)^38^.

### Proteomic profiling and identification

Proteomic profiling and identification in limonene treated *E. coli* was performed using 2D gel electrophoresis^39^. First dimension IEF done and strips focused on PROTEAN IEF unit (Bio-Rad) at 20 °C. Then strip were equilibrated and second dimension run on 12% SDS polyacrylamide gel at a constant voltage of 100 V for 3–4 h followed by staining with coomassie brilliant blue R250 for the visualization of proteins. Images of gel were analyzed with the help of Chemidoc [BIO-RAD] and proteins with different intensities were analyzed using PDQuest Advanced 2-D Gel Analysis software version 8.0.0 (Bio-Rad, Hercules, CA, USA).

### Chemotaxis

*Escherichia coli* was inoculated using capillary tube on nutrient agar plates containing 0.3% agar supplemented with limonene at sub–inhibitory concentration. Separate plate inoculated with glucose was taken as control. Following incubation, growth over agar surface away from inoculation point considered positive^40^.

### Inhibition of biofilm formation

Aliquot (100 µL) of overnight broth culture of *E. coli* adjusted to 0.1OD at 625 nm was treated with limonene (1XMIC and 2XMC) and its ability to form biofilm was observed after staining with 0.1% Crystal violet solution for 15 min followed by ethanol treatment with observation made at 590 nm^41^.

### Data analysis

The zone of inhibition data was subjected to Analysis of variance (ANOVA) and F-test at 0.1% level of significance. Tukey’s multiple comparison test was performed as post-hoc test after one-way ANOVA.

## Conclusion

Limonene qualified as broad-spectrum antibacterial agent. The series of events traced for its cidal property were chemotaxis, enzyme inhibition and regulation, disruption of translation in protein synthesis, alteration and damage of outer membrane, inhibition of cell wall synthesis, depolarization of inner membrane, inhibition of nucleic acid function and synthesis and leakage of cellular contents. The unique nature of limonene with multiple targets is definitely a promising antibacterial agent against multi-drug resistant bacterial pathogens.

## Supplementary Information

Supplementary Information.
