# Supplementary material for: Strategic approach of multifaceted antibacterial mechanism of limonene traced in Escherichia coli
Source: Sci Rep. 2021 Jul 5;11:13816. doi: 10.1038/s41598-021-92843-3 (PMC8257740; doi:10.1038/s41598-021-92843-3)
Supplement: Supplementary file 1 — Supplementary Information. [file 41598_2021_92843_MOESM1_ESM.pdf]

Title: Strategic approach of multifaceted antibacterial mechanism of limonene traced in *Escherichia coli*

Running Title: Antibacterial mechanism of Limonene

Authors: Akshi Gupta[1], Ebenezer Jeyakumar[2] and Rubina Lawrence[3]*

* corresponding author

Akshi Gupta[1]
Department of Industrial Microbiology
Jacob Institute of Biotechnology and Bioengineering
Sam Higginbottom University of Agriculture, Technology and Sciences
Prayagraj (Allahabad), Uttar Pradesh, India – 211007

Email: akshi.gupta.88@gmail.com

Dr. Ebenezer Jeyakumar[2]
Department of Industrial Microbiology
Jacob Institute of Biotechnology and Bioengineering
Sam Higginbottom University of Agriculture, Technology and Sciences
Prayagraj (Allahabad), Uttar Pradesh, India – 211007

Email: ebenezer.jeyakumar@gmail.com

Corresponding author:

Prof. (Dr.) Rubina Lawrence[3]*
Department of Industrial Microbiology
Jacob Institute of Biotechnology and Bioengineering
Sam Higginbottom University of Agriculture, Technology and Sciences
Prayagraj (Allahabad), Uttar Pradesh, India – 211007

Email: rubinalawrence@gmail.com

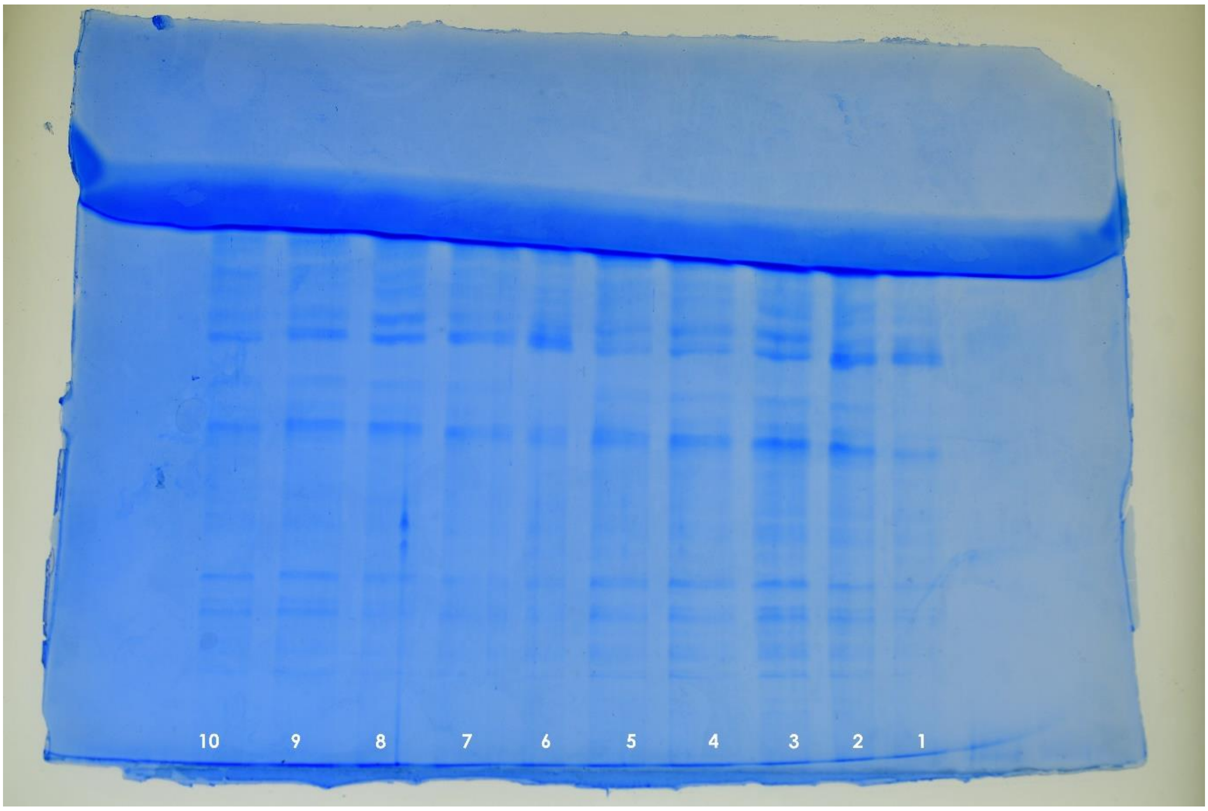

Figure 6 original

Fig 6: SDS-PAGE of proteins released from *Escherichia coli* into supernatant on treatment with limonene at 1XMIC and 2XMIC concentration

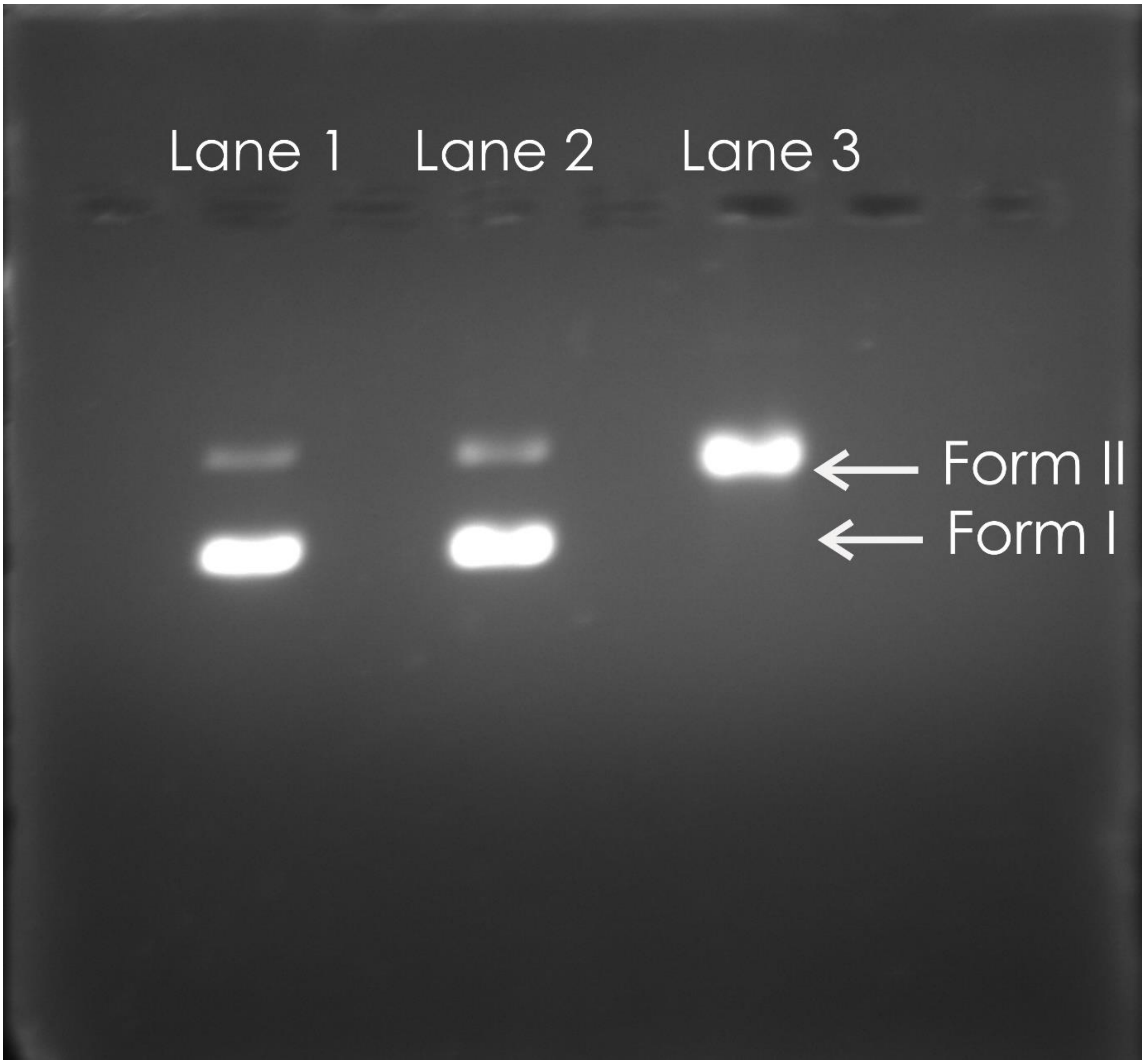

Figure 8 labelled

Fig. 8. Modification of gel electrophoretic mobility of pUC19 plasmid DNA after treatment with Limonene. Lane 1- Negative control, Lane 2- Pure plasmid, Lane 3- Limonene (1XMIC)

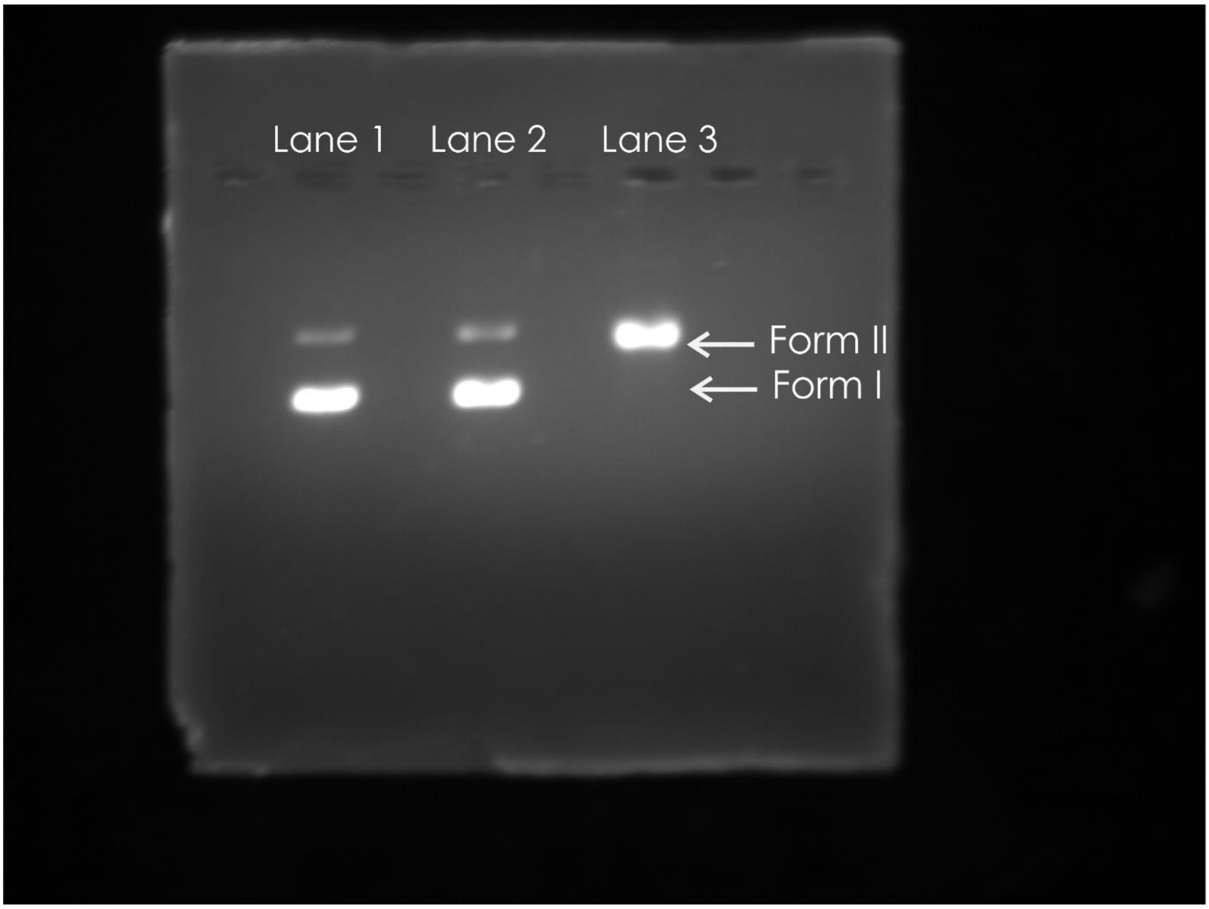

Figure 8 original

Fig. 8. Modification of gel electrophoretic mobility of pUC19 plasmid DNA after treatment with Limonene. Lane 1- Negative control, Lane 2- Pure plasmid, Lane 3- Limonene (1XMIC)

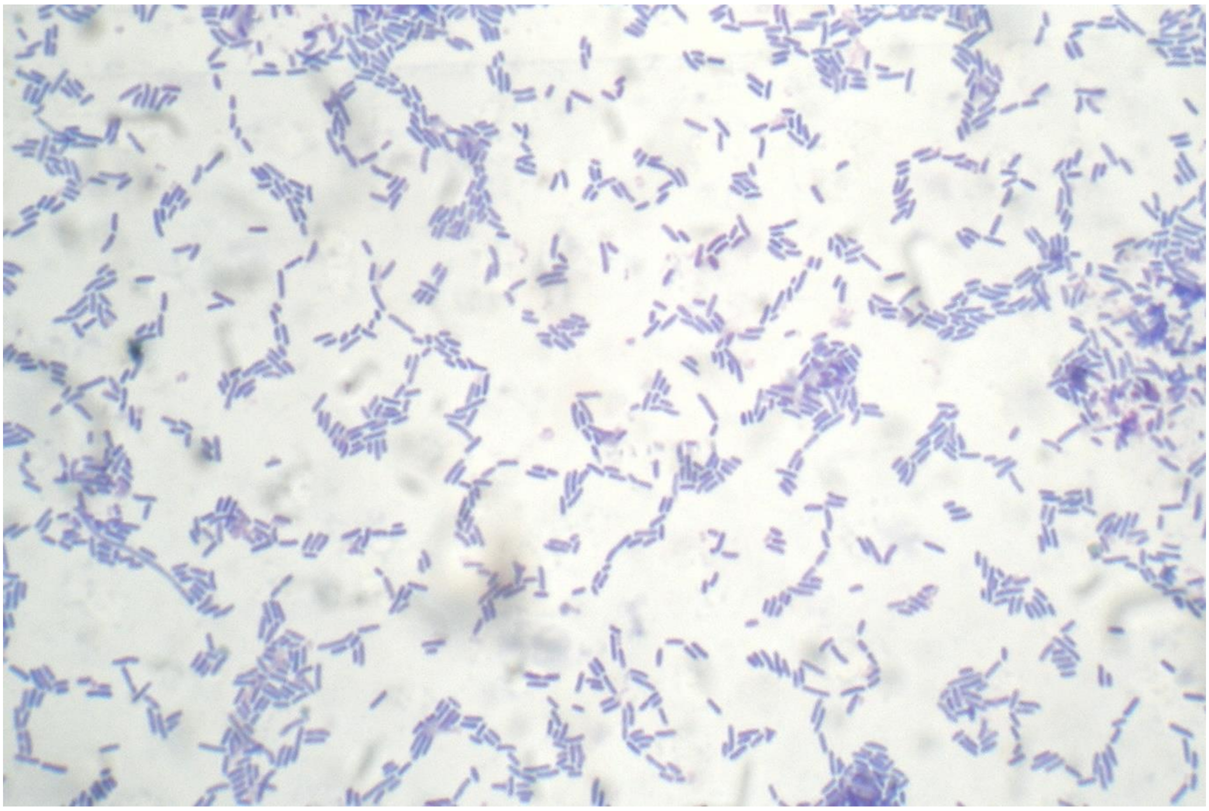

Figure 9a control untreated

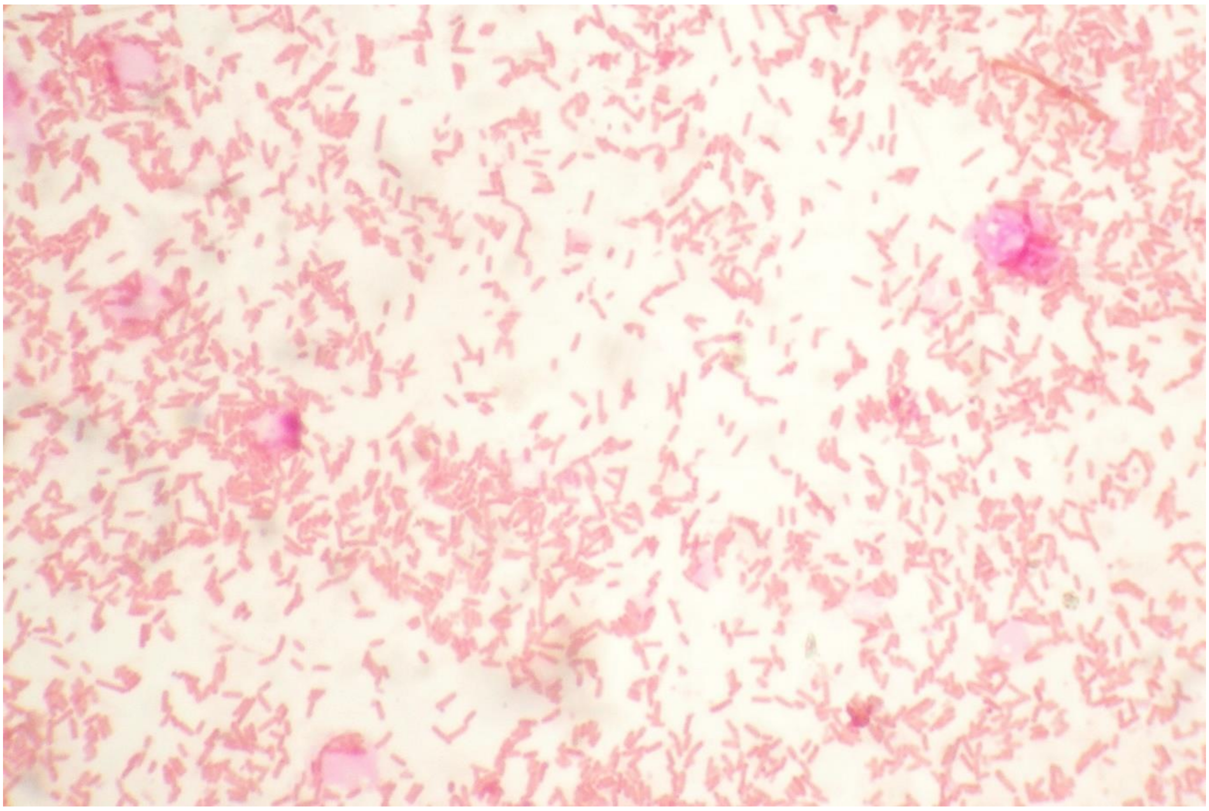

Figure 9b limonene treated

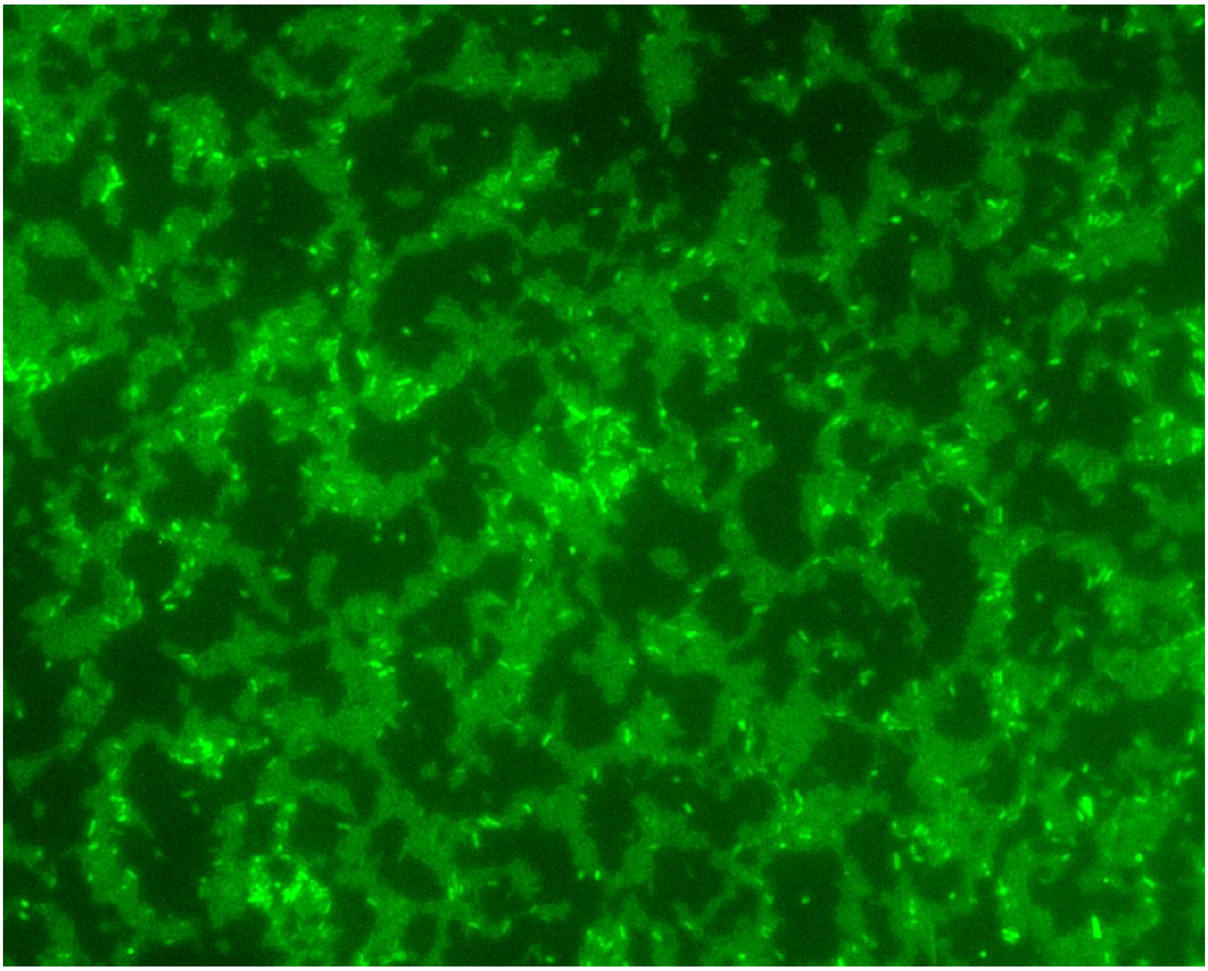

Figure 9c Untreated control

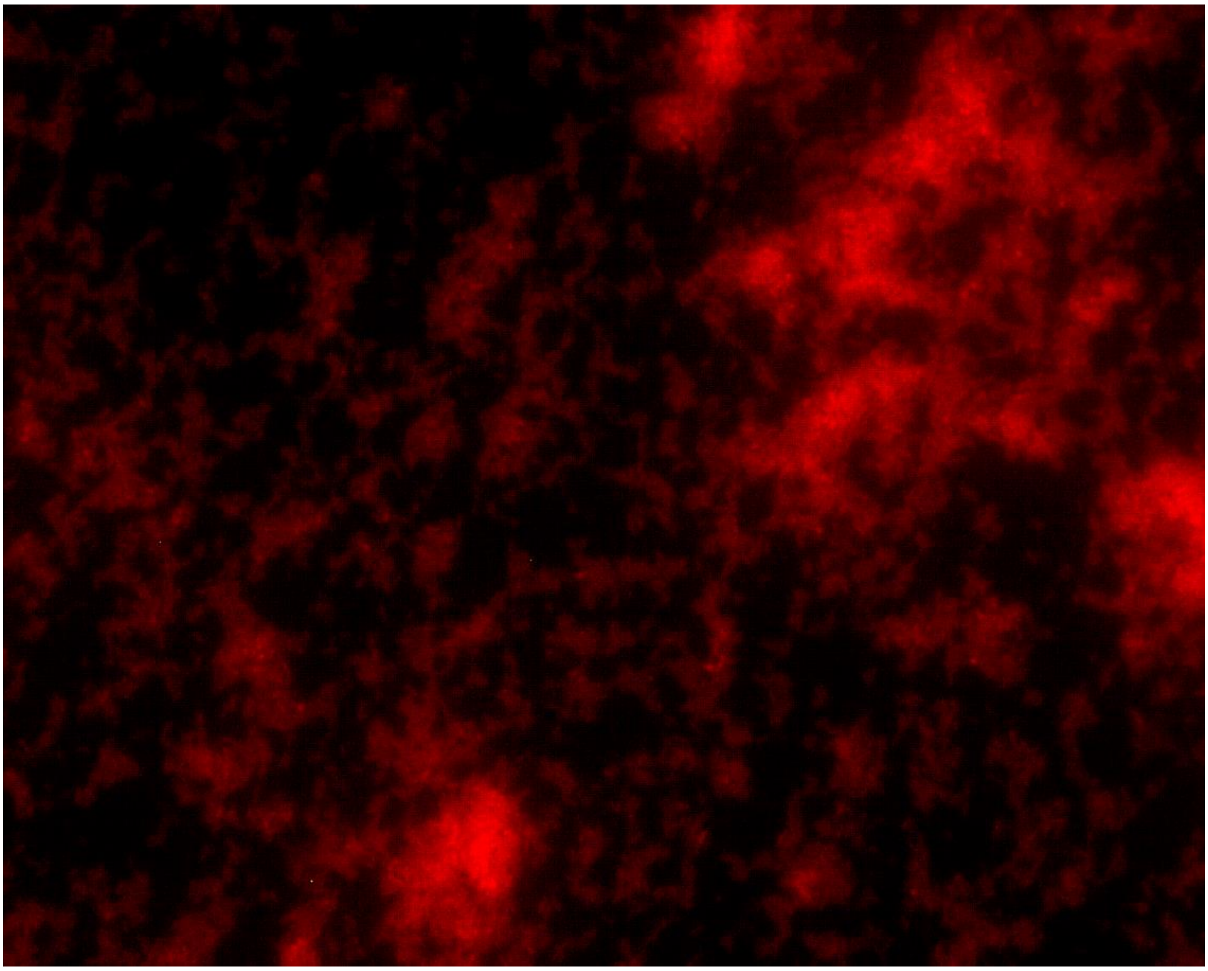

Figure 9d limonene Treated

Fig 9. Micrographs of untreated control and limonene treated (1XMIC) *E. coli* cells using Compound microscopy (a and b) fluorescent microscopy (c and d)
